# αTAT1 controls longitudinal spreading of acetylation marks from open microtubules extremities

**DOI:** 10.1038/srep35624

**Published:** 2016-10-18

**Authors:** Nathalie Ly, Nadia Elkhatib, Enzo Bresteau, Olivier Piétrement, Mehdi Khaled, Maria M. Magiera, Carsten Janke, Eric Le Cam, Andrew D. Rutenberg, Guillaume Montagnac

**Affiliations:** 1 Inserm U1170, Gustave Roussy Institute, Université Paris-Saclay, Villejuif, France; 2 CNRS UMR8126, Gustave Roussy Institute, Université Paris-Saclay, Villejuif, France; 3 CNRS UMR1186, Gustave Roussy Institute, Université Paris-Saclay, Villejuif, France; 4 Institut Curie, CNRS UMR3348, PSL Research University, Centre Universitaire, Orsay, France; 5 Department of Physics and Atmospheric Science, Dalhousie University, Halifax, NS, B3H 4R2, Canada

## Abstract

Acetylation of the lysine 40 of α-tubulin (K40) is a post-translational modification occurring in the lumen of microtubules (MTs) and is controlled by the α-tubulin acetyl-transferase αTAT1. How αTAT1 accesses the lumen and acetylates α-tubulin there has been an open question. Here, we report that acetylation starts at open ends of MTs and progressively spreads longitudinally from there. We observed acetylation marks at the open ends of *in vivo* MTs re-growing after a Nocodazole block, and acetylated segments growing in length with time. Bias for MTs extremities was even more pronounced when using non-dynamic MTs extracted from HeLa cells. In contrast, K40 acetylation was mostly uniform along the length of MTs reconstituted from purified tubulin *in vitro.* Quantitative modelling of luminal diffusion of αTAT1 suggested that the uniform acetylation pattern observed *in vitro* is consistent with defects in the MT lattice providing lateral access to the lumen. Indeed, we observed that *in vitro* MTs are permeable to macromolecules along their shaft while cellular MTs are not. Our results demonstrate αTAT1 enters the lumen from open extremities and spreads K40 acetylation marks longitudinally along cellular MTs. This mode of tip-directed microtubule acetylation may allow for selective acetylation of subsets of microtubules.

## Results and Discussion

Microtubules (MTs) are dynamic polymers composed of αβ-tubulin dimers that assembled into hollow tubes. In most eukaryotic cells, MTs can undergo post-translational modifications (PTMs) that modify their properties and functions^1^. Acetylation of the lysine 40 of α-tubulin (K40) is a common PTM that is catalysed by the α-tubulin acetyl-transferase αTAT1 and is associated with stable, long-lived MTs^2^,^3^,^4^. Remarkably, K40 acetylation occurs in the lumen of MTs^5^,^6^ and is the only such PTM that we know of ref. 1. Supporting this, Szyk *et al*. recently used *in vitro* approaches to demonstrate that αTAT1 enters into and diffuses within the MT lumen^7^. However, Szyk *et al*. also suggested that fast diffusivity of αTAT1 leads to stochastic acetylation that occurs uniformly along the length of MTs. This was in marked contrast with earlier *in vivo* observations of discrete acetylated segments along MTs^8^,^9^,^10^ progressively elongating with time^11^. More recently, several groups have reported that the acetylated segments were predominately associated with the ends of MTs *in vivo*^3^,^12^. Thus, reported observations *in vivo* do not match the proposed model of uniformly distributed acetylated K40 marks based on experiments performed with *in vitro* MTs^7^.

**Figure 1:**
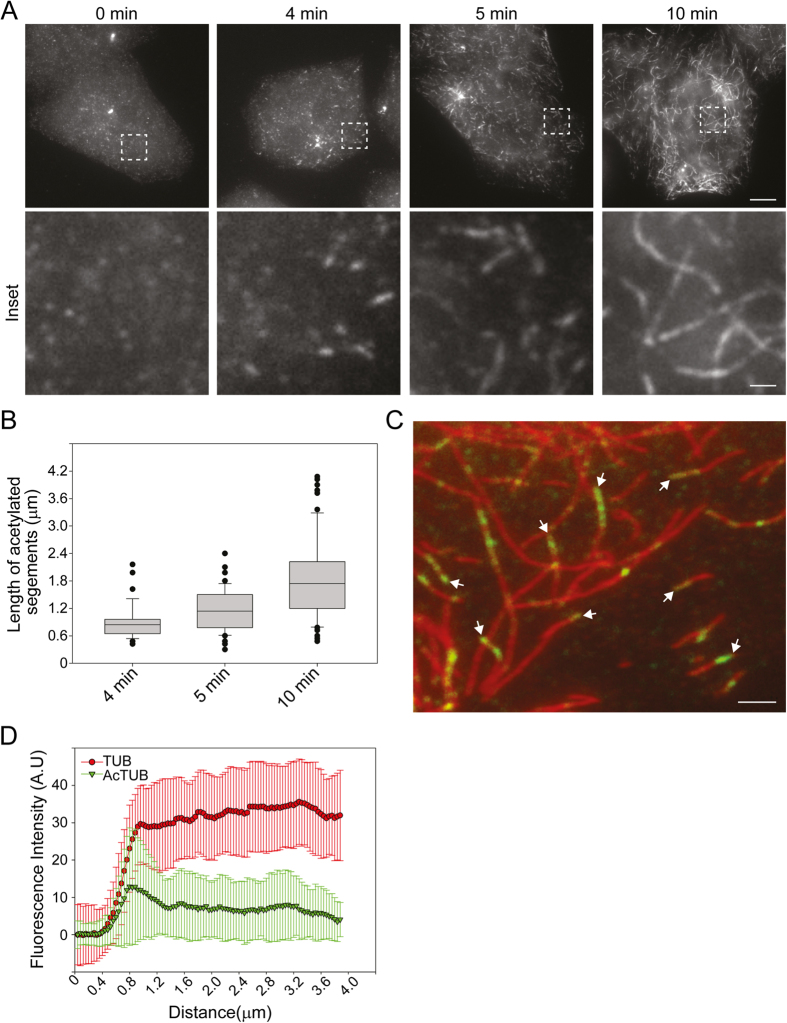
Preferential acetylation of MT ends *in vivo*. (**A**) HeLa cells recovering from a Nocodazole block were fixed at the indicated time point after washing out the drug and then stained for K40 acetylated tubulin. Scale bar: 10 μm. Insets corresponding to boxed regions are shown. Scale bar: 2 μm. (**B**) Box plot of acetylated segments length at the indicated time points. Data are represented as mean ± S.E.M. (**C**) HeLa cells recovering from a Nocodazole block were fixed at 5 min after washing out the drug and stained for total tubulin (red) and K40 acetylated tubulin (green). Arrows indicate acetylated MT extremities. Scale bar: 2 μm. (**D**) Average fluorescence intensity distribution of total tubulin staining (red) and K40 acetylated tubulin (green) along MTs as in C (see Experimental Procedures section). Data are represented as mean ± S.E.M.

To understand how acetylated K40 marks spreading occurs *in vivo*, we first analyzed acetylation dynamics in HeLa cells. In order to synchronize acetylation events, HeLa cells were subjected to complete MT depolymerisation by a prolonged treatment with Nocodazole before being allowed to reassemble MTs after washing out the drug. As previously reported, we observed that acetylated K40 segments became detectable as early as 4 minutes after Nocodazole washout (Fig. 1A). The length of these acetylated segments progressively increased with time (Fig. 1A,B). Careful examination of the distribution of acetylated segments visible at 5 minutes after washout revealed that acetylated K40 marks were frequently, but not always, associated with MT extremities (Fig. 1C). Measurements of the fluorescence intensity distribution along the long axis of MTs also showed an average enrichment of acetylated K40 marks at the extremities of MTs (Fig. 1D).

Our observation of a preference for acetylation at MT extremities agrees with earlier reports *in vivo*^3^,^12^ and suggests that αTAT1 accesses the lumen from the open ends of MTs and starts acetylating at those extremities. However, we also observed acetylation segments far from *in vivo* microtubule ends (Fig. 1C). While this might denote alternative lateral αTAT1 entry sites, we hypothesize instead that the extremely dynamic nature of microtubules *in vivo* allows unacetylated extensions to grow past acetylated K40-positive extremities, leaving acetylated segments behind. Indeed, microtubule polymerization is much faster than acetylation spreading (in the order of ~10 μm/min while acetylated segments elongate only 2 μm in 8 min as measured here). In this case, non-dynamic MTs should exhibit more acetylation segments at MT ends.

**Figure 2:**
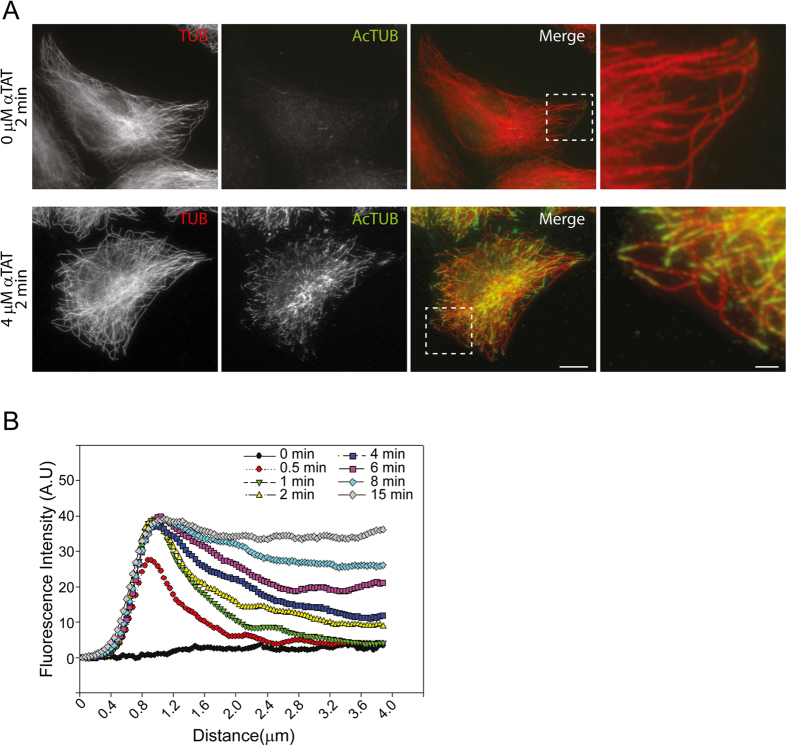
Longitudinal spreading of acetylated K40 marks from the ends of *ex vivo* MTs. (**A**) αTAT1-knockdowned HeLa cells were Triton-extracted in the presence of Taxol and incubated for 2 min without (upper panels) or with 4 μM recombinant αTAT1 (lower panels) before being fixed and stained for total (red) and acetylated tubulin (green). Scale bar: 10 μm. Insets corresponding to boxed regions are shown. Scale bar: 2 μm. (**B**) Average fluorescence intensity distribution of K40 acetylated tubulin along MTs (MTs tip at x = 1 μm) after indicated time period incubation with 4 μM recombinant αTAT1 as in A. Data are represented as mean. The S.E.M was omitted for clarity.

To test this hypothesis, we used αTAT1-knockdown HeLa cells extracted by a brief immersion in a MT stabilizing buffer containing Triton. In these conditions, Taxol-stabilized *ex vivo* cellular MTs were readily visible and were negative for anti-acetylated K40 staining (Fig. 2A, upper panels). *Ex vivo* MTs were then incubated with 4 μM of a recombinant catalytic domain of mouse αTAT1 (residues 1–193) in the presence of Acetyl-CoA for different time periods. Acetylated segments of MTs became visible as early as 30 s after addition of the recombinant enzyme and these segments were found to grow longer with time (Supplementary Figure 1), similarly to our *in vivo* observations. We then analysed the distribution of acetylated segments along MTs and observed that virtually all acetylated segments detected after a 2 min incubation period with the enzyme were located at the ends of individual MTs, with no detectable staining in other MT regions (Fig. 2A, lower panels). At the earliest time point analysed (30 s), small acetylated K40 segments were similarly found at the open extremity of MTs (Supplementary Figure 2). These tip-located acetylated segments grew in length with time, and by 15 min, most visible MTs were acetylated along their entire length (Supplementary Figure 2).

Measurements of fluorescence intensity showed that while total tubulin staining was constant along the length of *ex vivo* MTs (Supplementary Figure 3A), a strong bias for the extremities was observed for the acetylated K40 signal (Fig. 2B). We also noted a time-dependent progressive increase of staining intensity near the tips along with a progressive longitudinal (axial) spreading of acetylated K40 marks (Fig. 2B). We obtained similar results when using *ex vivo* MTs that were not stabilized with Taxol (Supplementary Figure 3B). In these latter conditions, the maximum intensity of acetylated K40 staining at the extremity was lower than in Taxol-stabilized *ex vivo* MTs. This may be due to a slow depolymerisation of MT ends leading to a partial loss of acetylation signal. Also, we performed acetylation assays on Taxol-stabilized *ex vivo* MTs by using a lower concentration (0.4 μM) of recombinant αTAT1. While the kinetics of acetylation was reduced, we observed the same preference for MT extremities and a progressive spreading of acetylated K40 marks from the ends (Supplementary Figure 3C). Together, our analysis unambiguously demonstrates that acetylation spreads progressively, longitudinally along *ex vivo* MTs, from their open extremities. Our data also suggest that acetylated segments observed far from MT ends *in vivo* most likely results from the dynamicity of MTs.

**Figure 3:**
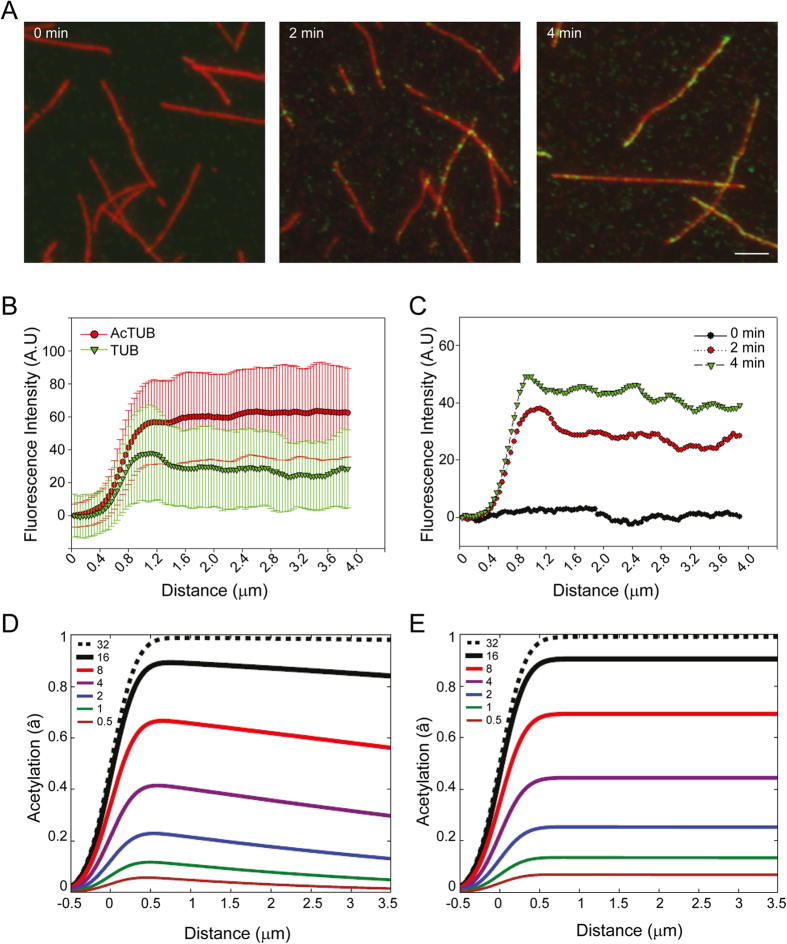
Stochastic acetylation of microtubules assembled *in vitro*. (**A**) MTs assembled *in vitro* from HeLa cell-purified tubulin dimers were incubated in the presence of 4 μM recombinant αTAT1 for the indicated time period before being fixed and stained for total tubulin (red) and acetylated K40 tubulin (green). Scale bar: 5 μm. (**B**) Average fluorescence intensity distribution of total tubulin staining (red) and acetylated K40 tubulin (green) along MTs (MTs tip at x = 1 μm) after a 2 min incubation period with recombinant αTAT1 as in A. Data are represented as mean ± S.E.M. (**C**) Average fluorescence intensity distribution of acetylated K40 tubulin along MTs (MTs tip at x = 1 μm) after a 0 (black), 2 (red) or 4 (green) min incubation period with recombinant αTAT1 as in A. Data are represented as mean. The S.E.M was omitted for clarity. (**E**,**D**) Theoretical average acetylation marks distribution along MTs (MTs tip at x = 1 μm) after the indicated incubation period with 4 μM αTAT1 without (**E**) or with (**D**) allowed lateral access. Data were convoluted with a 250 nm width Gaussian point-spread-function (PSF) to better qualitatively compare with the microscopy results. The color-coded legend indicates the time in minutes since entry into the unacetylated lumen begins.

Because this conclusion disagrees with reports of uniform acetylation *in vitro*^7^, we next decided to analyse acetylation dynamics in Taxol-stabilized MTs assembled *in vitro* from purified HeLa cells tubulin dimers. Because HeLa tubulin dimers are poorly acetylated, no acetylated K40 marks were observed immediately after polymerization *in vitro* (Fig. 3A, left panel). However, after a 2 minutes incubation period in the presence of 4 μM recombinant αTAT1, we observed a punctuated acetylated K40 staining with an approximately random distribution along the length of MTs (Fig. 3A, middle panel). After a 4 minutes incubation period with the enzyme, the acetylated K40 signal was more intense but still distributed randomly along the length of the MTs. Careful examination revealed that extremities were often positive for acetylated K40 marks (Fig. 3A), though this was less striking than *in vivo* (Fig. 1C). The average fluorescence intensity distribution also showed a slight enrichment of acetylated K40 marks at open extremities as compare to other regions of MTs (Fig. 3C). We obtained similar results when using MTs assembled *in vitro* from purified bovine brain tubulin (not shown). In agreement with observations by Szyk *et al*., we concluded that acetylation occurs randomly (uniformly) along the length of *in vitro* MTs^7^. This is in marked contrast with our *in vivo* and *ex vivo* observations and therefore suggests that *in vitro* and cellular MTs may differ in their intrinsic properties.

Structural defects in the MT lattice, such as missing tubulin dimers or abrupt variations of protofilament number have been reported in *in vitro* assembled MTs^13^,^14^. It was recently proposed that stress accumulation at these lattice defects can results in the formation of larger damages that self-repair rapidly^14^,^15^. Indeed, transient opening of the MT lattice resulting from separation between protofilaments have been hypothesized to provide direct access to the MT lumen in a proposed “breathing model”^16^,^17^. To explore the hypothesis that the differences we observed were due to transient holes or defects on the sides of *in vitro* MTs, we have developed a mathematical model of αTAT1 diffusion within MTs followed by local acetylation by αTAT1. We have included both lateral access (due to holes), and access from MT extremities, followed by longitudinal diffusion as characterized by Szyk *et al*.^7^ (see Experimental Procedures section for details). When lateral access was not allowed, we observed that acetylation marks spreads longitudinally from the open MT extremities (Fig. 3D). This is in qualitative agreement with our *ex vivo* results. However, when lateral access was implemented in the model using αTAT1 residence times determined by Szyk *et al*.^7^, acetylation was uniform along the length of MTs (Fig. 3E). Thus, our modelling captures the qualitative difference between the mostly uniform acetylation *in vitro* and the progressive acetylation *ex vivo* and suggests that the shaft of *in vitro* reconstituted MTs is permeable to αTAT1 while the one of cellular MTs is not.

With our quantitative model of longitudinal diffusion, local acetylation, and possible lateral entry we observed that tip-directed acetylation is consistent with the measured luminal diffusivity of αTAT1^7^, together with a moderate acetylation rate and vanishing lateral entry. Conversely, we showed that uniform acetylation is consistent with rapid lateral entry (with respect to the times at which measurements are taken) independent of the other parameters. Are other parameterizations, perhaps without lateral entry, consistent with the observed phenomenology? Without lateral entry, tip-directed acetylation should be observed at earlier times in longer MT. The earliest reported uniform acetylation was at 15s in 10 μm *in vitro* MT^7^, which we found (data not shown) requires luminal diffusivities at least 5 times larger than measured by Szyk *et al*. if uniformity of αTAT1 within 20% of the maximum is required. For 20 μm long MT, at least 20 times larger diffusivities are needed. However, such large luminal diffusivities preclude the tip-directed acetylation we observe *in vivo*. Since we have no reason to expect luminal diffusivities to hugely differ between *in vivo* and *in vitro* MT, we conclude that uniform acetylation observed for *in vitro* MT requires rapid lateral access of αTAT1.

**Figure 4:**
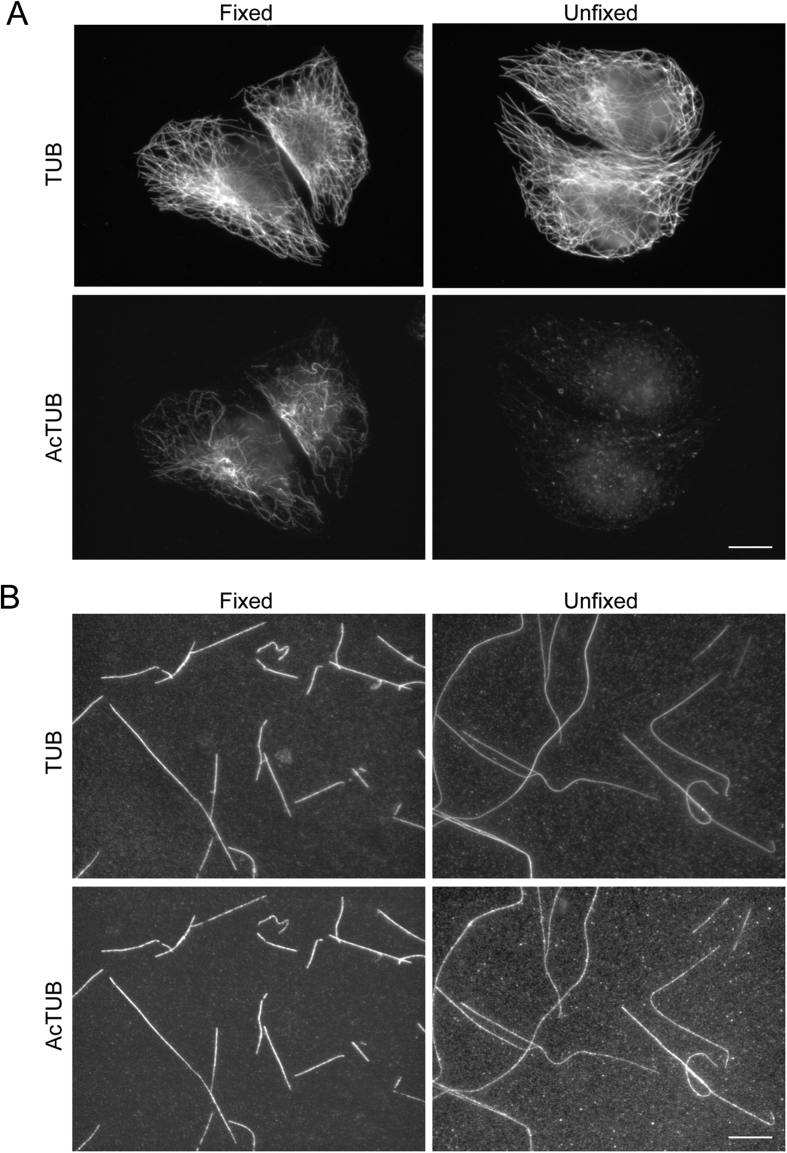
MTs assembled *in vitro* are permeable to anti-acetylated K40 antibody. (**A**) HeLa cells were Triton-extracted in the presence of Taxol and then fixed or not with MetOH as indicated before being stained for total (TUB) and acetylated tubulin (AcTUB). Scale bar: 10 μm. (**B**) MTs assembled *in vitro* from acetylated bovine brain-purified tubulin dimers were stabilized with Taxol and then fixed or not with MetOH as indicated before being stained for total (TUB) and acetylated tubulin (AcTUB). Scale bar: 10 μm.

If defects or holes in lattice allow αTAT1 to access the lumen of MTs assembled *in vitro*, the same could be true for other macromolecules. It has been reported that antibodies targeting luminal epitopes cannot access to the lumen of unfixed cellular MTs^18^. Indeed, while fixed, Triton-extracted HeLa cell MTs showed a classical anti-acetylated K40 staining, only a background staining was detectable in unfixed conditions (Fig. 4A). In sharp contrast, anti-acetylated K40 staining was visible along both fixed and unfixed *in vitro* MTs (Fig. 4B). We concluded that, in unfixed conditions, the anti-acetylated K40 antibody can access the lumen of *in vitro* but not cellular MTs. Our data suggest that the shaft of *in vitro* MTs is leaky and allows lateral access of macromolecules to the lumen. This agrees with both our modelling, and the observed uniform acetylation patterns of *in vitro* MTs.

It has been suggested that αTAT1 could bind to the outside of MTs and that this could facilitate targeting the enzyme to the lumen^19^. Indeed, we observed by Atomic Force Microscopy that αTAT1 binds to the exterior face of *in vitro* MTs (Supplementary Figure 4). This exterior binding could enhance αTAT1 entry to the lumen through holes or defects in the lattice of MTs polymerized *in vitro*, or to the open ends of MTs polymerized *in vivo*. This raises the possible that the reported scanning behaviour of individual αTAT1 molecules does not occur exclusively in the lumen of MTs as previously suggested^7^ but also on the outer surface, facilitating αTAT1 entry in the lumen. Such surface scanning behaviour would not change our results or conclusions, which relate to the luminal availability and acetylation activity of αTAT1.

To conclude, our data suggest that *in vitro* assembled MTs presents holes or defects along their shaft that allow lateral entry of αTAT1 to the lumen. The nature of such holes and why *in vitro* MTs would display more holes as compare to cellular MTs is not clear. One possibility is that MTs growing in the cellular context would be protected from developing or acquiring lateral access-promoting defects. Along this line, dislocation defects due to abrupt transition in the number of protofilaments have been reported to be twice as common *in vitro* as compare to *in vivo* conditions^13^. This raises the possibility that growth conditions for *in vitro* MT could be adjusted to reduce the number of holes or defects. Alternatively, microtubule-associated proteins (MAPs) might block lateral holes in cellular MTs, preventing αTAT1 access to the lumen at these sites. In this case, appropriate MAPs would need to be included in *in vitro* acetylation assays to recapitulate *in vivo* results.

Our results demonstrate that *in vitro* reconstituted MTs do not recapitulate the properties of cellular MTs and we urge great caution in studying the effects of MT acetylation with such MTs. Based on the apparent stochastic acetylation pattern *in vitro*, it was proposed that αTAT1 controls a selective, age-dependent MT acetylation^7^. In contrast to this model, the major finding of our work is that αTAT1 controls the longitudinal spreading of acetylation marks from open MT extremities in physiological conditions. In our model, the selective acetylation of a subset of MTs that is observed *in vivo* is not primarily controlled by the stability of this subset but rather by the chance that a given MT would acquire αTAT1 at its extremities. MTs have been shown to contact αTAT1-rich structures like clathrin-coated pits and focal adhesions, two types of structures that mostly accumulate at the front of migrating cells^12^,^20^,^21^. We propose that multiple contacts between MTs and these structures would enhance αTAT1 acquisition by front-oriented MTs leading to the progressive acetylation of this MT subset from their open extremities. This selective, tip-oriented acetylation mechanism has important consequences since cell-front oriented acetylated MTs are instrumental in controlling directional cell migration^12^.

## Methods

### Cell culture

HeLa cells (a gift from A. Dautry, Institut Pasteur, Paris, France) were grown in DMEM, high glucose, GlutaMax™ supplement media, supplemented with 10% fetal calf serum at 37 °C in 5% CO_2_.

### Antibodies and reagents

Mouse monoclonal anti-K40 acetylated tubulin (clone 6–11B1) was purchased from Sigma. Recombinant humanised anti-α-tubulin (clone F2C-hFc2) and Cy3-conjugated anti-human antibodies were purchased from the antibody platform of the Institut Curie (Paris, France). DyLight488-conjugated anti-mouse antibodies were from Jackson Immunoresearch. Nocodazole was purchased from Sigma.

### RNA interference

For siRNA depletion, HeLa cells were plated at 20% confluence and treated with the αTAT1 siRNA (25 nM; ON-TARGETplus SMARTpool, Dharmacon, Lafayette, CO, USA) using RNAiMAX transfection reagent according to the manufacturer’s instruction (Invitrogen, Carlsbad, CA). Protein depletion was maximal after 72 h of siRNA treatment as shown by immunofluorescence analysis with anti-acetylated K40 antibodies. Sequences of αTAT1 siRNAs: 5′-GUAGCUAGGUCCCGAUAUA-3′, 5′-GAGUAUAGCUAGAUCCCUU-3′, 5′-GGGAAACUCACCAGAACGA-3′, 5′-CUUGUGAGAUUGUCGAGAU-3′.

### DNA constructs and protein purification

DNA sequence encoding residues 1–193 (catalytic domain) of mouse αTAT1 was obtained by PCR by using cDNA of C-terminally GFP-tagged mMEC17 as a template (Montagnac *et al*., Nature, 2013). PCR fragment with engineered flanking restriction sites was subcloned into the multi-cloning sites of pGEX4T1 (Amersham Pharmacia Biotech) to encode in-frame fusion proteins with the amino-terminal GST-tag. The construct was verified by double-stranded DNA sequencing. Purification of the recombinant protein was performed using standard protocols as described^12^. HeLa cell tubulin and bovine brain tubulin were purified as described^22^.

### Indirect immunofluorescence

For analysis of acetylation dynamics with *in vivo* microtubules, HeLa cells plated onto coverslips were incubated in the presence of 10 μM Nocodazole for 5 hours to induce microtubule depolymerisation. Cells were then washed three times in complete medium to washout Nocodazole and allow microtubules to re-grow. Cells were then fixed at indicated time points after washout in ice-cold methanol and processed for immunofluorescence microscopy by using anti-α-tubulin and anti-acetylated K40 antibodies.

For *ex vivo* acetylation assay, cells were washed in PEM buffer (80 mM PIPES, 1 mM EGTA, 1 mM MgCl_2_, pH 6, 8) before microtubules extraction in PEM buffer containing 1% Triton X-100 and 10 μM Taxol. Acetylation reactions were performed in PEM buffer containing 10 μM Taxol and the indicated concentration of recombinant αTAT1 in presence of 50 μM acetyl coenzyme A (Sigma) at 37 °C and stopped at indicated time points by fixation with ice cold methanol before being processed for immunofluorescence microscopy by using anti-α-tubulin and anti-acetylated K40 antibodies.

For the *in vitro* microtubule acetylation assay, 25 μM HeLa cell-purified tubulin was incubated in PEM buffer supplemented with 15% glycerol and 1 mM GTP, for 40 min at 37 °C to promote polymerization. Microtubules were then diluted in PEM progressively supplemented with 10 μM taxol (Sigma). Acetylation reactions were performed in PEM-Taxol buffer with 4 μM recombinant αTAT, in presence of 50 μM acetyl coenzyme A (Sigma) at 37 °C and stopped at indicated time points by fixation with 2% glutaraldehyde. Microtubules were adsorbed on poly-L-lysine coated coverslips and processed for immunofluorescence microscopy by using anti-α-tubulin and anti-acetylated K40 antibodies.

For experiments with unfixed microtubules, Triton-extracted HeLa cell microtubules or microtubules assembled *in vitro* from purified bovine brain tubulin were prepared as described above and incubated with the anti-acetylated K40 antibody for 30 min at room temperature. For *in vitro* microtubules, this incubation step was performed in suspension before microtubules were washed and spotted on a glass coverslip. Microtubules were then processed for immunofluorescence microscopy as described above except that microtubules were not fixed.

Cells or microtubules were imaged with the 100× objective of a wide-field microscope DM6000 B/M (Leica Microsystems) equipped with a CCD CoolSnap HQ camera (Roper Scientific) and steered by Metamorph 7 (Molecular Devices). All pictures shown are representative of at least three independent experiments.

### Analysis of fluorescence distribution along microtubules

Analysis of total tubulin or acetylated K40 tubulin signal distribution along microtubules was performed using Metamorph 7 (Molecular Devices) software by drawing a 4 μm long linescan along the long axis of unambiguously individual microtubules, starting 1 μm in front of the visible tip. Total or acetylated K40 background fluorescence values were subtracted for each individual microtubules. At least 100 microtubules from 3 independent experiments were analysed for each time points. Data were pooled and expressed as mean fluorescence ± S.E.M. over distance. S.E.M. was omitted in some figures for clarity.

### Modelling Methods

From the one-dimensional diffusion equation for the time-evolution of a linear density *ρ*(*x*, *t*) of αTAT1 in the MT lumen,


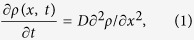


the solution^23^ is


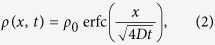


where the position *x* ≥ 0 is measured from the end of the MT and the time *t* ≥ 0 is measured from when αTAT1 is added. Here *ρ*_0_ represents the steady-state luminal concentration of αTAT1 that results from whatever bulk concentration is imposed from outside the MT. This differs slightly from the model of Szyk *et al*., since they used a fixed length MT open at both ends^7^. In addition, Szyk *et al*. did not consider acetylation or lateral entry of αTAT1 in their quantitative model^7^.

We model the acetylation as a local density of available sites *a*(*x*, *t*) that are acetylated by locally available αTAT1, so that


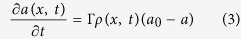


where Γ is our acetylation rate. This is easily solvable in closed form, and defining 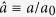 and 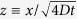 we obtain


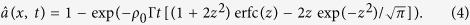


To model the effects of lateral entry of αTAT1 into the MT lumen, through transient or static holes, we assume uniform entry so that


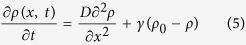


where the rate *γ* captures the size, density, frequency, and lifetime of holes that allow αTAT1 entry. The closed-form solution is


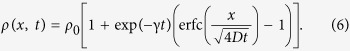


We see that as *γ* → ∞, we immediately impose the steady-state luminal concentration *ρ* = *ρ*_0_. When *γ* > 0, we solve for the time-dependent acetylation numerically to obtain 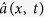-note that the spatial-dependence of 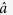 will no longer be simply in terms of 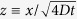.

To evaluate our analytical and numerical results, we need to determine the diffusivity *D*, the lateral entry rate *γ*, and the combination 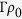. Since acetylation is reported in arbitrary units, we do not need an explicit value of *a*_0_–i.e. we plot 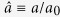.

Szyk *et al*. determined *D* = 0.27 *μm*^2^/*s* from tracking of individual fluorescently-labeled αTAT1 in the MT lumen^7^. *D* is approximately 100x smaller than the geometrically hindered diffusivity *D*_*hindered*_ = 26.3 *μm*^2^/*s* expected for a particle the size of αTAT1 in the tube-like MT geometry^7^. This difference is due to the reversible binding of αTAT1 to the MT^24^, with *D* = α*D*_*hindered*_. The correction factor α is the duty-factor, or the proportion of time that αTAT1 is unbound and diffusing. The 100x reduced *D* results from αTAT1 being almost always immobilized with α ≈ 0.01. Crucially, a key assumption is that the binding kinetics are “faster than diffusion transport rates”^24^. From the video microscopy of Szyk *et al*., no immobilized αTAT1 is apparent by eye. This implies very fast binding/unbinding within the lumen, with 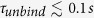, which is consistent with the assumptions of Odde and Crank for length-scales greater-than 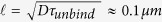.

Szyk *et al*. determined a lifetime *τ* = 1.5 *s* of αTAT1 “interaction” in the MT lumen (see their Fig. 5c). However, *τ* cannot be a transition between *D* and *D*_*hindered*_, since *D* = 0.27 *μm*^2^/*s* already includes the effects of binding/unbinding and we have already seen that binding and unbinding are relatively fast. Instead, we interpret *τ* as a lifetime of residence in the MT lumen. We believe *τ* represents the rate of escape from the MT lumen, and so also determines the lateral entry rate 
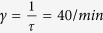
. Finally, Shida *et al*. determined *k*_*cat*_ = 615±34×10^−6^ *s*^−1^ for 1 *μM* bulk αTAT1^4^. We take Γ*ρ*_0_ = 4 *k*_*cat*_ = 0.15/*min*, corresponding to our 4 *μM* bulk αTAT1.

### Atomic Force Microscopy

As described above microtubules polymerization was promoted by incubating tubulin in PEM buffer supplemented with 15% glycerol and 1 mM GTP, for 40 min at 37 °C to. Microtubules were then diluted in PEM supplemented with 10 μM taxol. Experiments with αTAT were carried out in PEM-Taxol buffer with 400 nM recombinant αTAT1 at 37 °C.

5 μl of microtubules aliquot (with and without αTAT1) were deposited on mica surface pretrated with 100 μM spermidine^25^ and incubated for typically 20 s, which allows the diffusion of microtubules on the surface and their adsorption^26^. The mica surface was then plunged for 30s in uranyl acetate solution (0.02% w/v) for microtubules fixation on mica surface. Finally, the sample was rinsed with pure water (Millipore) and dried with a filter paper. AFM imaging was carried out in Tapping Mode, with a Multimode system (Bruker) operating with a Nanoscope V controller (Bruker). We used silicon AC200TS cantilevers (Olympus) with resonance frequencies around 150 kHz. All images were collected at a scan frequency of 1 Hz and a resolution of 2048 × 2048 pixels. Images were analysed with Nanoscope V software, and a third-order polynomial function was used to remove the background.

## Additional Information

**How to cite this article**: Ly, N. *et al*. αTAT1 controls longitudinal spreading of acetylation marks from open microtubules extremities. *Sci. Rep.* **6**, 35624; doi: 10.1038/srep35624 (2016).

## Supplementary Material

Supplementary Information

## References

[b1] Song Y. & Brady S. T. Post-translational modifications of tubulin: pathways to functional diversity of microtubules. Trends in cell biology 25, 125–136 (2015). 25468068 10.1016/j.tcb.2014.10.004 PMC4344850

[b2] Perdiz D., Mackeh R., Pous C. & Baillet A. The ins and outs of tubulin acetylation: more than just a post-translational modification? Cellular signalling 23, 763–771 (2011). 20940043 10.1016/j.cellsig.2010.10.014

[b3] Akella J. S. . MEC-17 is an alpha-tubulin acetyltransferase. Nature 467, 218–222 (2010). 20829795 10.1038/nature09324 PMC2938957

[b4] Shida T., Cueva J. G., Xu Z., Goodman M. B. & Nachury M. V. The major alpha-tubulin K40 acetyltransferase alphaTAT1 promotes rapid ciliogenesis and efficient mechanosensation. Proc Natl Acad Sci USA 107, 21517–21522 (2010). 21068373 10.1073/pnas.1013728107 PMC3003046

[b5] Nogales E., Whittaker M., Milligan R. A. & Downing K. H. High-resolution model of the microtubule. Cell 96, 79–88 (1999). 9989499 10.1016/s0092-8674(00)80961-7

[b6] Soppina V., Herbstman J. F., Skiniotis G. & Verhey K. J. Luminal localization of alpha-tubulin K40 acetylation by cryo-EM analysis of fab-labeled microtubules. PloS One 7, e48204 (2012). 23110214 10.1371/journal.pone.0048204 PMC3482196

[b7] Szyk A. . Molecular basis for age-dependent microtubule acetylation by tubulin acetyltransferase. Cell 157, 1405–1415 (2014). 24906155 10.1016/j.cell.2014.03.061 PMC4726456

[b8] Piperno G., LeDizet M. & Chang X. J. Microtubules containing acetylated alpha-tubulin in mammalian cells in culture. J Cell Biol 104, 289–302 (1987). 2879846 10.1083/jcb.104.2.289 PMC2114420

[b9] Webster D. R. & Borisy G. G. Microtubules are acetylated in domains that turn over slowly. J Cell Sci 92 (Pt 1), 57–65 (1989). 2674164 10.1242/jcs.92.1.57

[b10] Wilson P. J. & Forer A. Effects of nanomolar taxol on crane-fly spermatocyte spindles indicate that acetylation of kinetochore microtubules can be used as a marker of poleward tubulin flux. Cell motility and the cytoskeleton 37, 20–32 (1997). 9142436 10.1002/(SICI)1097-0169(1997)37:1<20::AID-CM3>3.0.CO;2-L

[b11] Bulinski J. C., Richards J. E. & Piperno G. Posttranslational modifications of alpha tubulin: detyrosination and acetylation differentiate populations of interphase microtubules in cultured cells. J Cell Biol 106, 1213–1220 (1988). 3283150 10.1083/jcb.106.4.1213 PMC2115029

[b12] Montagnac G. . alphaTAT1 catalyses microtubule acetylation at clathrin-coated pits. Nature 502, 567–570 (2013). 24097348 10.1038/nature12571 PMC3970258

[b13] Chretien D., Metoz F., Verde F., Karsenti E. & Wade R. H. Lattice defects in microtubules: protofilament numbers vary within individual microtubules. J Cell Biol 117, 1031–1040 (1992). 1577866 10.1083/jcb.117.5.1031 PMC2289483

[b14] Schaap I. A., Carrasco C., de Pablo P. J., MacKintosh F. C. & Schmidt C. F. Elastic response, buckling, and instability of microtubules under radial indentation. Biophysical journal 91, 1521–1531 (2006). 16731557 10.1529/biophysj.105.077826 PMC1518621

[b15] Schaedel L. . Microtubules self-repair in response to mechanical stress. Nature materials 14, 1156–1163 (2015). 26343914 10.1038/nmat4396 PMC4620915

[b16] Diaz J. F., Barasoain I. & Andreu J. M. Fast kinetics of Taxol binding to microtubules. Effects of solution variables and microtubule-associated proteins. J Biol Chem 278, 8407–8419 (2003). 12496245 10.1074/jbc.M211163200

[b17] Diaz J. F., Valpuesta J. M., Chacon P., Diakun G. & Andreu J. M. Changes in microtubule protofilament number induced by Taxol binding to an easily accessible site. Internal microtubule dynamics. J Biol Chem 273, 33803–33810 (1998). 9837970 10.1074/jbc.273.50.33803

[b18] Draber P., Draberova E., Linhartova I. & Viklicky V. Differences in the exposure of C- and N-terminal tubulin domains in cytoplasmic microtubules detected with domain-specific monoclonal antibodies. J Cell Sci 92 (Pt 3), 519–528 (1989). 2480356 10.1242/jcs.92.3.519

[b19] Howes S. C., Alushin G. M., Shida T., Nachury M. V. & Nogales E. Effects of tubulin acetylation and tubulin acetyltransferase binding on microtubule structure. Mol Biol Cell 25, 257–266 (2014). 24227885 10.1091/mbc.E13-07-0387 PMC3890346

[b20] Kaverina I., Krylyshkina O. & Small J. V. Microtubule targeting of substrate contacts promotes their relaxation and dissociation. J Cell Biol 146, 1033–1044 (1999). 10477757 10.1083/jcb.146.5.1033 PMC2169483

[b21] Castro-Castro A., Janke C., Montagnac G., Paul-Gilloteaux P. & Chavrier P. ATAT1/MEC-17 acetyltransferase and HDAC6 deacetylase control a balance of acetylation of alpha-tubulin and cortactin and regulate MT1-MMP trafficking and breast tumor cell invasion. European journal of cell biology 91, 950–960 (2012). 22902175 10.1016/j.ejcb.2012.07.001

[b22] Barisic M. . Mitosis. Microtubule detyrosination guides chromosomes during mitosis. Science 348, 799–803 (2015). 25908662 10.1126/science.aaa5175 PMC4506776

[b23] Crank J. The Mathematics of Diffusion, 2nd edition (Oxford University Press, Oxford UK, 1975).

[b24] Odde D. Diffusion inside microtubules. European biophysics journal: EBJ 27, 514–520 (1998). 9760732 10.1007/s002490050161

[b25] Pastre D. . Anionic polyelectrolyte adsorption on mica mediated by multivalent cations: a solution to DNA imaging by atomic force microscopy under high ionic strengths. Langmuir: the ACS journal of surfaces and colloids 22, 6651–6660 (2006). 16831009 10.1021/la053387y

[b26] Hamon L., Curmi P. A. & Pastre D. High-resolution imaging of microtubules and cytoskeleton structures by atomic force microscopy. Methods in cell biology 95, 157–174 (2010). 20466134 10.1016/S0091-679X(10)95009-7

