# Supplementary material for: αTAT1 controls longitudinal spreading of acetylation marks from open microtubules extremities
Source: Sci Rep. 2016 Oct 18;6:35624. doi: 10.1038/srep35624 (PMC5067677; doi:10.1038/srep35624)
Supplement: Supplementary Information [file srep35624-s1.pdf]

αTAT1 controls longitudinal spreading of acetylation marks from open microtubules extremities

Nathalie Ly[1*], Nadia El Khatib[1*], Enzo Bresteau[1], Olivier Piétrement[2], Mehdi Khaled[3], Maria M. Magiera[4], Carsten Janke[4], Eric Le Cam[2], Andrew D Rutenberg[5,*] and Guillaume Montagnac[1,*]

Supplementary Information

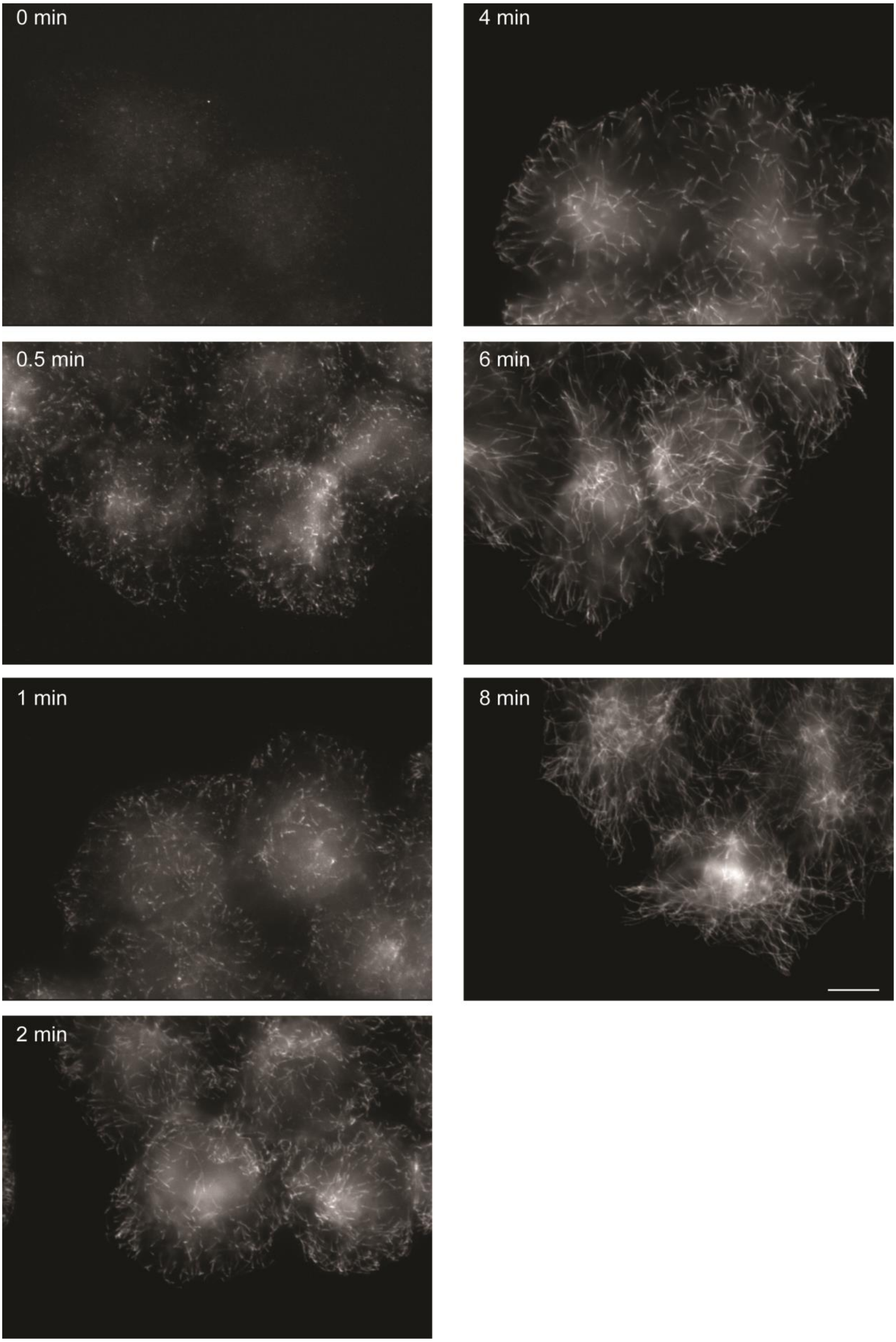
0 min
4 min
0.5 min
6 min
1 min
8 min
2 min

Figure S1

αTAT1-knockdown HeLa cells were extracted in the presence of Taxol and incubated for indicated time with 4 μM recombinant αTAT1 before being fixed and stained for acetylated tubulin. Scale bar: 10 μm.

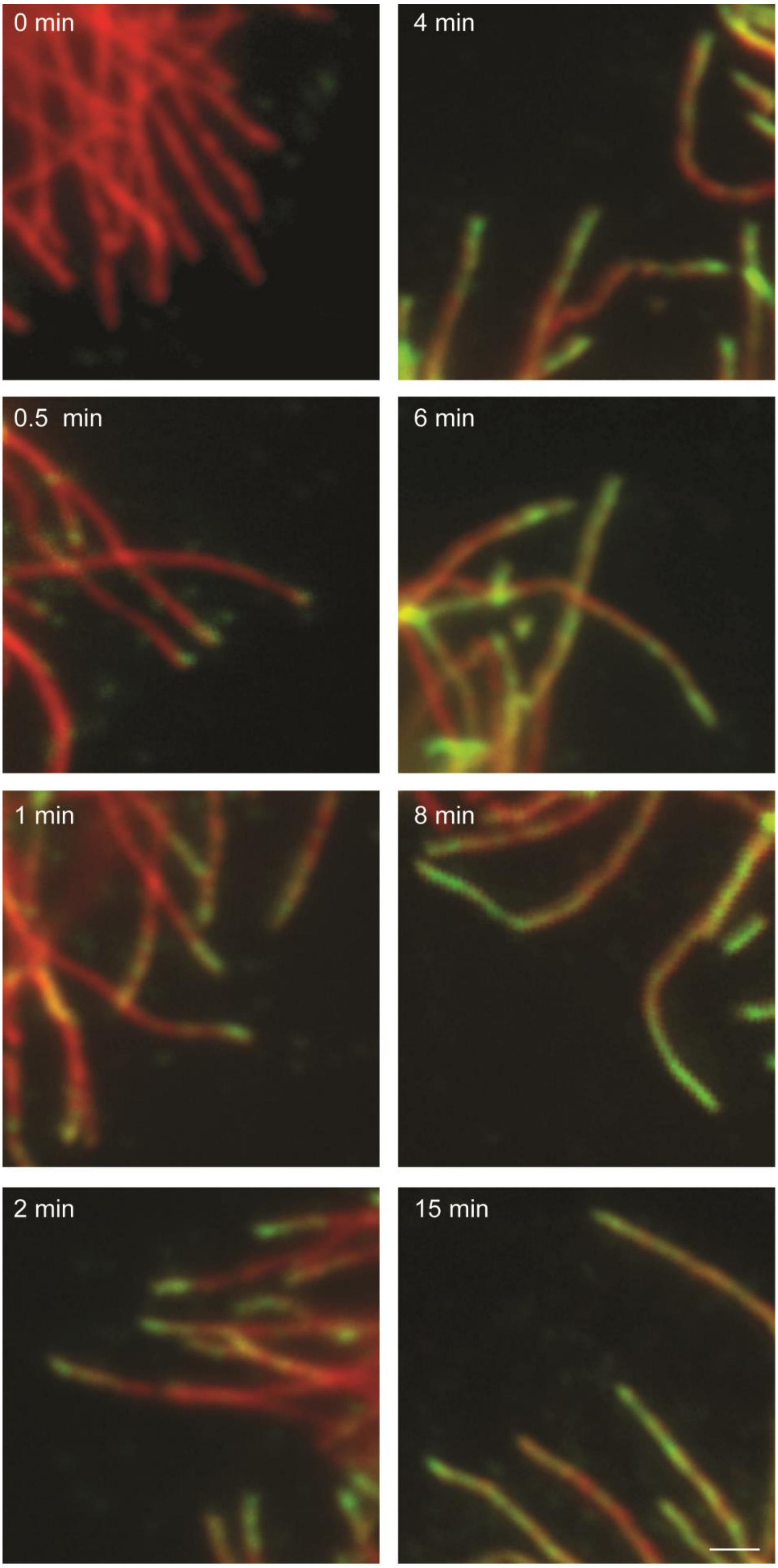
0 min
4 min
0.5 min
6 min
1 min
8 min
2 min
15 min

Figure S2

αTAT1-knockdown HeLa cells were extracted in the presence of Taxol and incubated for the indicated time with 4 μM recombinant αTAT1 before being fixed and stained for total (red) and acetylated tubulin (green). Scale bar: 1 μm.

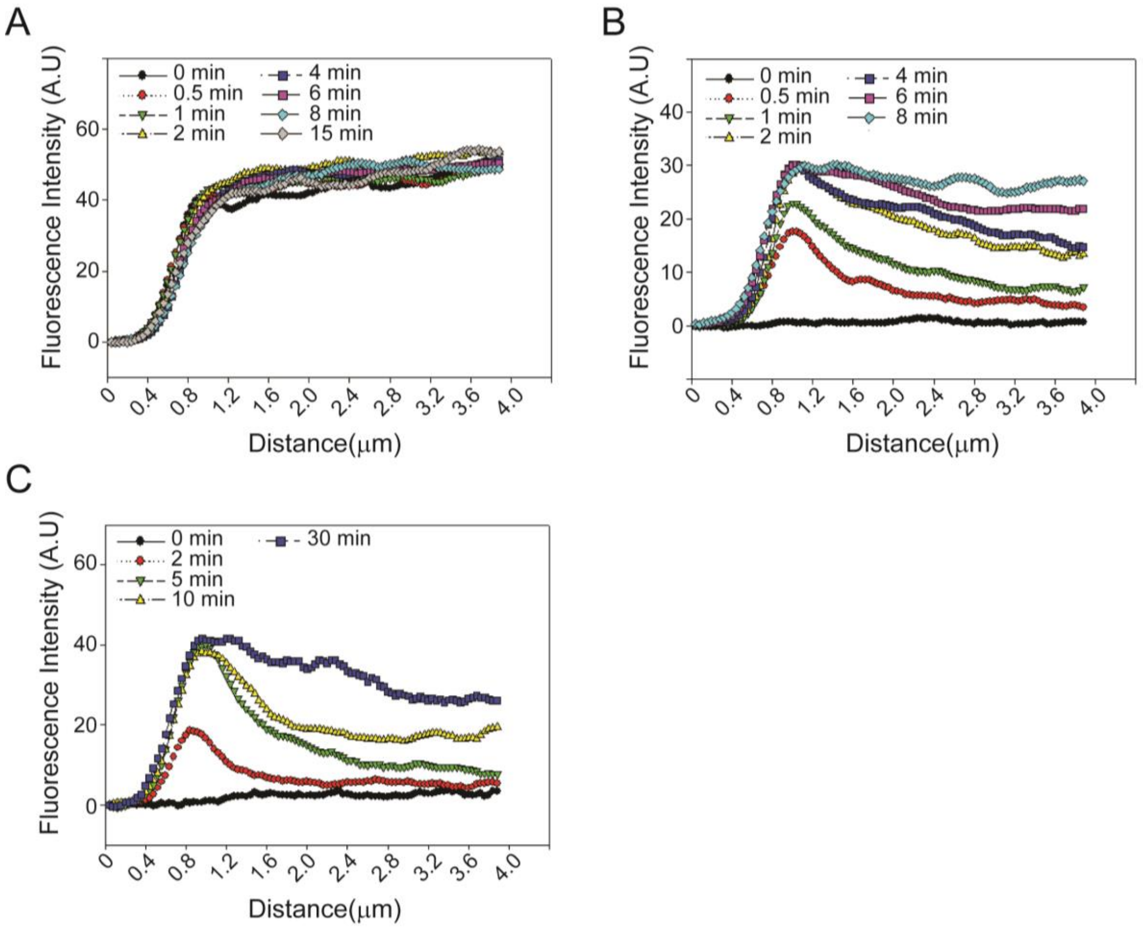

Figure S3

(**A**) Average fluorescence intensity distribution of total tubulin along MTs (MTs tip at x=1 μm) after indicated time period incubation with 4 μM recombinant αTAT1. Data are represented as mean. S.E.M was omitted for clarity.

(**B**) Average fluorescence intensity distribution of acetylated K40 tubulin along non-Taxol-stabilized MTs (MTs tip at x=1 μm) after indicated time period incubation with 4 μM recombinant αTAT1. Data are represented as mean. S.E.M was omitted for clarity.

(**C**) Average fluorescence intensity distribution of acetylated K40 tubulin along MTs (MTs tip at x=1 μm) after indicated time period incubation with 0.4 μM recombinant αTAT1. Data are represented as mean. S.E.M was omitted for clarity.

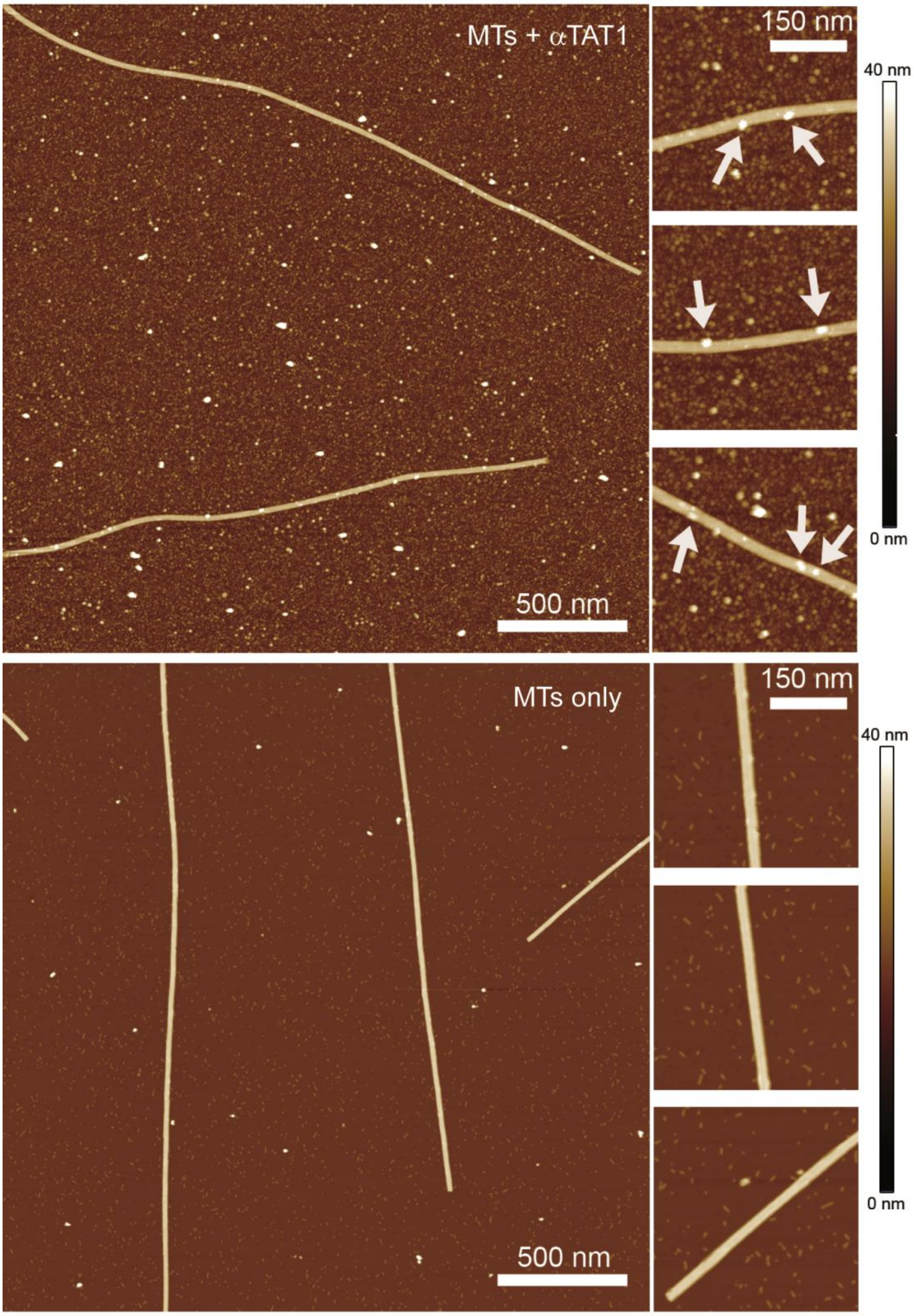

Figure S4

MTs assembled *in vitro* from HeLa cell-purified tubulin dimers were incubated in PEM buffer supplemented with 0.4 μM recombinant αTAT1 for 10 minutes (top panels) or not (bottom panel) before being processed for atomic force microscopy. Magnified pictures are shown on

the right. Arrows indicate αTAT1 on MTs. Scale bars are indicated. Height range is shown on the right.
